# Genomic analysis of childhood hearing loss in the Yoruba population of Nigeria

**DOI:** 10.1038/s41431-021-00984-w

**Published:** 2021-11-26

**Authors:** Adebolajo Adeyemo, Rabia Faridi, Parna Chattaraj, Rizwan Yousaf, Risa Tona, Samuel Okorie, Thashi Bharadwaj, Liz M. Nouel-Saied, Anushree Acharya, Isabelle Schrauwen, Robert J. Morell, Suzanne M. Leal, Thomas B. Friedman, Andrew J. Griffith, Isabelle Roux

**Affiliations:** 1 Institute of Child Health, College of Medicine, University of Ibadan, Ibadan, Nigeria; 2 Laboratory of Molecular Genetics, National Institute on Deafness and Other Communication Disorders (NIDCD), National Institutes of Health, Bethesda, MD 20892 USA; 3 Otolaryngology Branch, NIDCD, National Institutes of Health, Bethesda, MD 20892 USA; 4 Center for Statistical Genetics, Gertrude H. Sergievsky Center, Department of Neurology, Columbia University Medical Center, 630 W 168th St, New York, NY 10032 USA; 5 Genomics and Computational Biology Core, NIDCD, National Institutes of Health, Bethesda, MD 20892 USA; 6 Taub Institute for Alzheimer’s Disease and the Aging Brain, Columbia University Medical Center, 630 W 168th St, New York, NY 10032 USA; 7 Present Address: Department of Otolaryngology, College of Medicine, University of Tennessee Health Science Center, 910 Madison Avenue, Memphis, TN 38163 USA

**Keywords:** Genetics research, Medical genomics

## Abstract

Although variant alleles of hundreds of genes are associated with sensorineural deafness in children, the genes and alleles involved remain largely unknown in the Sub-Saharan regions of Africa. We ascertained 56 small families mainly of Yoruba ethno-lingual ancestry in or near Ibadan, Nigeria, that had at least one individual with nonsyndromic, severe-to-profound, prelingual-onset, bilateral hearing loss not attributed to nongenetic factors. We performed a combination of exome and Sanger sequencing analyses to evaluate both nuclear and mitochondrial genomes. No biallelic pathogenic variants were identified in *GJB2*, a common cause of deafness in many populations. Potential causative variants were identified in genes associated with nonsyndromic hearing loss (*CIB2, COL11A1*, *ILDR1*, *MYO15A*, *TMPRSS3*, and *WFS1*), nonsyndromic hearing loss or Usher syndrome (*CDH23*, *MYO7A*, *PCDH15*, and *USH2A*), and other syndromic forms of hearing loss (*CHD7*, *OPA1*, and *SPTLC1*). Several rare mitochondrial variants, including m.1555A>G, were detected in the gene *MT-RNR1* but not in control Yoruba samples. Overall, 20 (33%) of 60 independent cases of hearing loss in this cohort of families were associated with likely causal variants in genes reported to underlie deafness in other populations. None of these likely causal variants were present in more than one family, most were detected as compound heterozygotes, and 77% had not been previously associated with hearing loss. These results indicate an unusually high level of genetic heterogeneity of hearing loss in Ibadan, Nigeria and point to challenges for molecular genetic screening, counseling, and early intervention in this population.

## Introduction

Hearing loss (HL) is one of the most common sensory disorder worldwide. In high-income countries (HICs), the estimated prevalence of permanent bilateral HL of 40 dB HL or more is estimated to be between 1.33 and 1.5 per 1000 live births; 2.7 per 1000 by 5 years of age [for review [1, 2]]. HL seems to be more prevalent in Sub-Saharan Africa [3]. In Nigeria, the exact prevalence of HL (as defined by >40 dB HL) is unknown but has been estimated to be at least 19.2 per 1000 newborns [4–6].

Early childhood HL in HICs is thought to be primarily due to genetic factors and monogenic variants, although permanent profound HL can also be caused by environmental factors, or a combination of both [7, 8]. In Western Africa and Nigeria, environmental factors have been reported as frequent risk factors for HL [3, 9–12]. There are only a few published studies of genetic contributions to HL in Sub-Saharan African populations [13–15]. In many populations, pathogenic variants of *GJB2* [MIM: 121011] sometimes in *trans* with deletions in the *GJB2*/*GJB6* genomic region (del(*GJB6*-D13S1830), del(*GJB6*-D13S1854), and del(chr13:19,837,344-19,968,698) [16, 17] represent a common cause of genetic HL. Variants in these genes have rarely been found in individuals with HL from Sub-Saharan Africa or of Sub-Saharan African descent, except in Ghana where the pathogenic variant NM_004004.6:c.427C>T p.(Arg143Trp) of *GJB2* is prevalent [13]. Pathogenic variants in other genes associated with HL such as *SLC26A4* [MIM: 605646], *OTOF* [MIM: 603681], *HGF* [MIM: 142409], *MYO15A* [MIM: 602666], and *TMC1* [MIM: 606706] are found frequently in specific populations outside of Sub-Saharan Africa. It is still unclear whether pathogenic variants in these same genes or others are associated with HL in populations of Sub-Saharan Africa, and whether some of these pathogenic variants are common.

Nigeria is located in Sub-Saharan West Africa and is the most populous country in Africa with approximately 210 million inhabitants. Our cohort is comprised of families from Ibadan, Nigeria, with at least one member with nonsyndromic congenital or early childhood severe-to-profound HL without a known environmental cause. We used exome and Sanger sequencing analyses in these subjects to study nuclear and mitochondrial genes reported to be associated with human nonsyndromic or syndromic HL in other populations, and identify potential causative variants associated with HL in Yoruba ethnic group.

## Materials and methods

### Study approval

This study was approved by the University of Ibadan, University College Hospital Ethics Committee (UI/EC/15/0047), the Combined Neurosciences Institutional Review Board at the National Institutes of Health (01-DC-0229), and the Institutional Review Board of Columbia University (IRB-AAAS2343). All adult participants provided written informed consent. For minors, at least one parent provided consent and minors provided their assents.

### Subjects, recruitment, and ascertainment

Fifty-six families were studied, which included 70 individuals with HL and 102 individuals without HL (Supplementary Table 1). Probands were identified from government schools for the deaf or from the Deaf Community in Ibadan, southwest Nigeria. The majority of subjects were of the Yoruba ethnic origin, the most prevalent ethnic group in southwest Nigeria. All probands were of Black African ancestry with bilateral congenital or prelingual-onset nonsyndromic HL. Whenever possible, parents, siblings, and other relatives of probands were enrolled. Individuals with HL associated with obvious features of known syndromes or whose HL was likely secondary to a nongenetic etiology such as trauma, infection, metabolic, or immunologic disorders, or exposure to ototoxic agents such as noise or aminoglycoside antibiotics were excluded from the study. None of the probands had a reported history of dizziness or significant neonatal illness. Physical examinations were carried out by a physician (AA) to identify or rule out obvious syndromic features. Pure-tone audiometry was performed, in some cases, to confirm bilateral severe-to-profound sensorineural HL. Family history was obtained by interview of all participants.

### DNA sequencing analyses

**Fig. 1:**
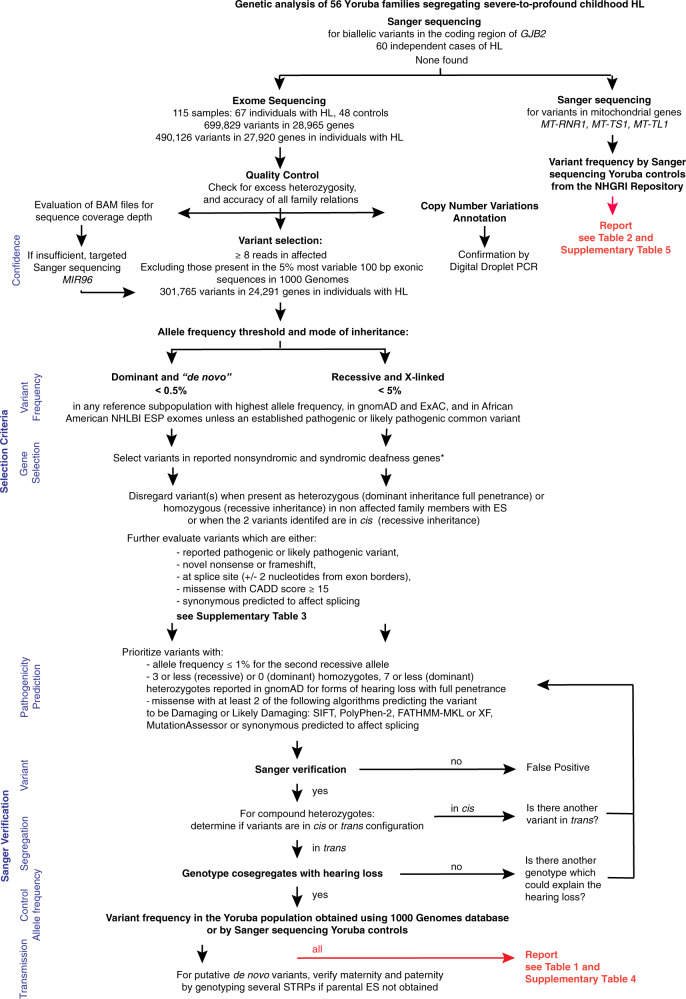
Experimental and analysis pipeline used to identify potential causative variants in reported genes associated with nonsyndromic and syndromic forms of hearing loss. Diagram shows the experimental strategy used for processing and analyzing the samples and data. Sanger sequencing was done on the gDNA of both parents if they had HL or on the gDNA of the proband with HL otherwise, for a total of 60 independent cases of HL. Variant counts are shown for the initial steps. The lists of genes included for these analyses are presented in Supplementary Information 2 and 3. *Full penetrance of HL was assumed unless reported otherwise for some genes associated with syndromic forms of HL. STRP short tandem repeat polymorphisms.

Genomic DNA (gDNA) was extracted from peripheral blood samples and processed as shown in Fig. 1. We first screened whole gDNA extract from one subject with HL from each family, or both parents if they both had HL (*N* = 60 independent cases tested), by Sanger sequencing the single protein-coding exon of *GJB2*, NM_000601.6:c.482+1986_1988delTGA and c.482+1991_2000delGATGATGAAA intronic deletions of *HGF* that are frequent in Asia [18] and the mitochondrial genes *MT-RNR1*, *MT-TS1*, and *MT-TL1* using custom designed primers (Supplementary Table 2). Exome sequencing (ES) was performed with gDNA samples from 67 individuals with HL and 48 family members without reported HL. ES libraries were prepared using a Nextera Rapid Capture Exome kit (Illumina, San Diego, CA, USA) and sequenced on an Illumina NextSeq500 instrument. The mean depth of coverage of the targeted coding regions was 44×. Due to insufficient coverage, microRNA *MIR96* [MIM: 611606, (*DFNA50*)] was Sanger-sequenced [19].

### Data analyses

Sequence data from exome libraries were mapped to the GRCh38 human reference genome using the bcbio-nextgen germline variant calling pipeline. See Supplementary Information 1 for all URLs and corresponding references. Reads were mapped using the Burrows–Wheeler Aligner (BWA-MEM), then remapped after removing duplicate data and recalibration with Genome Analysis Toolkit (GATK). Single-nucleotide variants (SNV) and insertion/deletions were detected using GATK-haplotypecaller, platypus, varscan, freebayes, and samtools variant callers. The final ensemble variant call file (vcf) required concordance between at least two callers. As a quality control, we checked the entire cohort for excess heterozygosity and verified the sex of each individual with exome data using PLINKv1.9. Family relationships were verified via both Identity-by-Descent sharing (PLINKv1.9) and Kinship-based INference for Gwas (KING) algorithm. Samples identified to have problems were removed from further exome data analysis. Individual vcf files were then analyzed using the Ingenuity Variant Analysis platform (IVA version 5.6, Qiagen, Hilden, Germany) using data from gnomAD v2.1.1, The Human Gene Mutation Database (HGMD) v2019.2, NCBI dbSNP v151, and Allele Frequency Community (AFC) v2019-09-25. The systematic review of the variants and prioritization of the predicted pathogenic, likely pathogenic, and variants of uncertain significance (VUS) with predicted deleterious effects segregating in each family was performed as shown in Fig. 1 and is summarized below. Copy number variants (CNVs) were assessed using the copy number inference from exome reads (CoNIFER) pipeline.

### Variant prioritization and verification

Single-nucleotide and insertion-deletion variants detected in at least eight reads from any one individual with HL, and absent from the top 5% of most variable 100-bp exonic sequences in 1000 Genomes database, were further analyzed. We first searched for variants in genes associated with nonsyndromic HL likely to be pathogenic (Supplementary Information 2) assuming complete penetrance. In Family 29, HL was present in both parents and children. In most families, only one individual with HL was present and the family history was limited. Variants were analyzed and evaluated according to several inheritance models (Fig. 1). In order to avoid missing prevalent and potentially enriched pathogenic alleles (founder variants), we used thresholds that were larger, by a factor of ten, than conventional thresholds for minor allele frequency for HL [20]. Assuming a model of autosomal dominant (AD) inheritance and searching for *de novo* variants, variants were only considered when: (1) they were already established to be pathogenic or likely pathogenic, (2) they were not detected in unaffected parents, or (3) when the ES data from one or both parents were not available, their minor allele frequency (MAF) was ≤0.5% in any reference subpopulation in gnomAD, ExAC, and in African (American) from the National Heart, Lung, and Blood Institute Exome Sequencing Project Exome Variant Server. In models of autosomal recessive (AR) and X-linked inheritance (XL), only variants with a frequency of less than 5% in the variant databases were initially considered, unless they were an established pathogenic or likely pathogenic variant. Variants detected in family members without reported HL that were either homozygous or heterozygous in *cis* without additional variant in *trans* were disregarded. Previously reported pathogenic variants, novel nonsense or frameshift variants, splice site variants within two nucleotides of intron-exon junctions, and missense variants with a combined annotation-dependent depletion (CADD) score of at least 15 were reported and further analyzed, as well as synonymous variants predicted to affect splicing (Supplementary Table 3). Variants were prioritized for further study when: (1) one of them had an allele frequency ≤1% (recessive model); (2) there were five or less (recessive model) or no (dominant model) individuals homozygous for the variant reported in gnomAD; and (3) if at least two algorithms predicted the variant to be damaging/deleterious or likely damaging/deleterious. The algorithms used were SIFT, PolyPhen-2, FATHMM-MKL or XF, MutationAssessor, MutationTaster, REVEL, and CADD (Supplementary Information 1). Evolutionary conservation of nucleotides was evaluated by PhyloP. The effects of variants on mRNA splicing were predicted by MaxEntScan, Human splice finder, BDGP Splice Site Prediction, and NetGene2.

In addition, a second and independent SNV/insertion/deletion annotation and prioritization analysis was performed with the same outcome, on the jointly called variants with GATK only, similar to what has been described in detail [21]. In short, variants were annotated using ANNOVAR, including prediction scores from dbnsfp35a and dbscSNV1.1, ClinVar, and several frequency databases such as gnomAD. Variants were considered further if either (1) they were reported pathogenic/likely pathogenic in ClinVar; or (2) they met filtering criteria including inheritance model (AR, AD, and XL [including *de novo*]), variant location (exonic and splice site), predicted effect (missense, nonsense, frameshift, and in-frame insertion/deletion, splicing and start and stop altering), and variant frequency (<0.5% MAF for AR and XL; <0.05% MAF for AD) [21].

Prioritized variants were validated via Sanger sequencing and further tested for co-segregation with HL in each family. Variant frequency in the Yoruba population was determined using Ensembl to access data from 108 Yoruba individuals from Ibadan, Nigeria (AFR YRI) available through 1000 Genomes. When a variant site was not evaluated due to insufficient read depth for example, we performed Sanger sequencing using gDNA samples from 118 unrelated Yoruba individuals (59 females and 59 males) from Ibadan, Nigeria (NHGRI Repository at the Coriell Institute for Medical Research Cat # MGP00013). Variants were then classified according to the American College of Medical Genetics and Genomics/Association for Molecular Pathology (ACMG/AMP) Guidelines for the interpretation of sequence variants in HL genes [22], taking into account data from ClinVar, the Deafness Variation Database, and HGMD, with two modifications. The genetic causes of HL have not yet been well characterized in the YRI population, and the information regarding variant MAF in this population is still limited, so we did not exclude any variant based on their “high” MAF. PP3 criterion was applied even if the REVEL score was below 0.7, if at least two of the algorithms used predicted that the variant was damaging or likely damaging (Fig. 1 and Table 1). In families with potential *de novo* variants, maternity and paternity were verified by genotyping short tandem repeat markers when no parental ES data were available. A similar analysis was performed for genes implicated in syndromic forms of HL assuming full penetrance unless otherwise reported [23].

**Table 1:** Likely causative variants identified by exome sequencing in genes reported to be associated with hearing loss.

Family	Individual with HL	Gene	Form of HL	Inheritance	Zygosity	HGVS transcript:nucleotide change	Predicted protein change	Splicing modification:MaxEntScan diff	SIFT function	PolyPhen-2 function prediction	MutationTaster	FATHMM-MKL or XF	MutationAssessor	Conservation based on phyloP	REVEL score	CADD score	MAF in YRI samples from the Coriell (Sanger sequencing)	MAF in YRI population (1000 genomes)	MAF in gnomAD in African/ African American population in percent	MAF in gnomAD (all populations) in percent	ACMG/AMP classification with HL specifications*
*Variants identified in genes associated with nonsyndromic forms of hearing loss*																					
**2**	**II.3, II.4**	***CIB2***	DFNB48	AR	Hom	NM_006383.4:c.556C>T	**p.(Arg186Trp)**	–	D	PbD	DC	B	M		0.418	34		0/216	0.052	0.005	Likely pathogenic
**57**	**I.2**	***ILDR1***	DFNB42	AR	Het	NM_001199799.2:c.775C>T	**p.(Arg259Ter)**	–			DC	B		C	–	42	0/236	ND	0	0.005	Pathogenic
		***ILDR1***		AR	Het	NM_001199799.2:c.9G>A	**p.(Trp3Ter)**	–			DC	B			–	25.9	0/236	ND	ND	ND	Pathogenic
**6**	**II.2**	***MYO15A***	DFNB3	AR	Het	NM_016239.4:c.4888C>T	**p.(Arg1630Cys)**	–	D	PbD	DC	P	H		0.62	28.6		4/216	0.856	0.091	VUS
		***MYO15A***		AR	Het	NM_016239.4:c.5777G>A	**p.(Arg1926His)**	–	D	PbD	DC	P	M	C	0.783	33		0/216	0.021	0.006	VUS
**13**	**II.6**	***MYO15A***	DFNB3	AR	Het	NM_016239.4:c.3196G>C	**p.(Ala1066Pro)**	–	D	B	Po	B	L		0.196	15.6		1/216	0.273	0.071	VUS
		***MYO15A***		AR	Het	NM_016239.4:c.7006C>T	**p.(Gln2336Ter)**	–			DC	B			–	35	0/236	ND	ND	ND	Likely pathogenic
**44**	**II.3**	***MYO15A***	DFNB3	AR	Het	NM_016239.4:c.4216G>A	**p.(Glu1406Lys)**	–	D	PbD	DC	P	H	C	0.95	32	0/234	ND	0	0.001	VUS
		***MYO15A***		AR	Het	NM_016239.4:c.6302T>C	**p.(Leu2101Pro)**	–	D	PbD	DC	P	M	C	0.836	32	0/236	ND	0.042	0.004	VUS
**40**	**II.1**	***TMPRSS3***	DFNB8/B10	AR	Hom	NM_024022.3:c.1363T>C	**p.(Ter455ArgextTer9)**	–			Po	P			–	<10		3/216	0.256	0.025	VUS
		***TMPRSS3***		AR	Hom	NM_024022.3:c.323-6G>A		4.423			DC	P			–	<10		0/216	0	0.0001	Pathogenic
**23**	**II.1**	***COL11A1***	DFNA37, Marshall syndrome, Stickler syndrome type II	AD	Het	NM_001854.4:c.1031C>T	**p.(Thr344Met)**	–	T	PbD		P	M	C	0.493	24.3		0/216	0.006	0.002	VUS
**32**	**II.1**	***COL11A1***	DFNA37, Marshall syndrome, Stickler syndrome type II	AD	Het	NM_001854.4:c.1314G>A	**p.(Met438Ile)**	–	T	PsD	DC	P	N	C	0.503	23.4	0/236	ND	ND	ND	VUS
		***COL11A1***			Het in trans - present in I.2 without HL	NM_001854.4:c.4049C>G	**p.(Ser1350Cys)**	–	T	PbD	DC	P	M	C	0.733	35	0/236	ND	ND	ND	VUS
**51**	**II.4**	***WFS1***	DFNA6/A14/A38, Wolfram-like syndrome (AD)	AD	Het	NM_006005.3:c.2029G>A	**p.(Ala677Thr)**	–	D	PsD	DC	P	M	C	0.73	23.5	0/236	ND	0	0.009	VUS
*Variants identified in genes associated with nonsyndromic forms of hearing loss or Usher syndrome*																					
**8**	**II.4, II.5**	***CDH23***	DFNB12, USH1D	AR	Compound Het	NM_022124.6:c.3176A>T	**p.(Asp1059Val)**	–		PbD	DC	P	H	C	0.965	34	0/236	ND	ND	ND	Likely pathogenic
		***CDH23***		AR	Compound Het	NM_022124.6:c.7872+1G>A		8.182			DC	Ph		C	–	32	0/236	ND	ND	ND	Pathogenic
**21**	**II.2**	***CDH23***	DFNB12, USH1D	AR	Het	NM_022124.6:c.271C>T	**p.(Gln91Ter)**	–			DC	B		C	–	40	0/236	ND	ND	ND	Pathogenic
		***CDH23***		AR	Het in *cis* with c.8177C>T	NM_022124.6:c.5237G>A	**p.(Arg1746Gln)**	–	T	B	DC	P	N	C	0.354	23.5	0/236	ND	0.008	0.007	Pathogenic
		***CDH23***		AR	Het in *cis* with c.5237G>A	NM_022124.6:c.8177C>T	**p.(Pro2726Leu)**	0.683	D	PsD	DC	P	N	C	0.268	27.6	0/236	ND	0	0.001	Likely pathogenic
**60**	**I.2**	***CDH23***	DFNB12, USH1D	AR	Compound Het	NM_022124.6:c.5505G>A	**p.(Met1835Ile)**	0.46	T	B	DC	P	N		0.1	22.4		0/216	0.169	0.05	VUS
		***CDH23***		AR	Compound Het	NM_022124.6:c.9726del	**p.(Ser3243ProfsTer5)**	–			DC			C	–	36	2/236	ND	0.102	0.009	VUS
		***OTOF***	DFNB9	AR	Compound Het	NM_194248.3:c.245G>A	**p.(Arg82His)**	–	T	PsD	DC	P	L	C	0.23	23.9		1/216	0.6245	0.068	VUS
		***OTOF***		AR	Compound Het	NM_194248.3:c.3917A>C	**p.(Lys1306Thr)**	–	T	B	DC	P	L	C	0.454	23.4		0/216	0.279	0.021	VUS
**56**	**I.1**	***CDH23***	DFNB12, USH1D	AR	Het	NM_022124.6:c.2560C>T	**p.(Arg854Cys)**	–		PbD	DC	P	L		0.189	31	0/236	ND	0.004	0.001	VUS
		***CDH23***		AR	Het	NM_022124.6:c.3074G>A	**p.(Gly1025Asp)**	–		PsD	DC	P	M	C	0.925	33		0/216	1.177	0.35	VUS
**56**	**I.2**	***MYO7A***	DFNB2, USH1B	AR	Het unlikely to be in *cis* with the other variants	NM_000260.4:c.133-3C>A		4.21			DC	Ph		C	–	21.4	0/236	ND	ND	ND	VUS
		***MYO7A***		AR	Het	NM_000260.4:c.6355C>A	**p.(Gln2119Lys)**	−0.92	T	PbD	DC	P	M	C	0.767	34	0/236	ND	ND	ND	VUS
		***MYO7A***		AR	Het	NM_000260.4:c.5522C>T	**p.(Thr1841Met)**	–	D	PbD	DC	P	M	C	0.815	24.9	0/236	ND	0.01	0.006	VUS
**5**	**III.2, III.3, III.4**	***PCDH15***	DFNB23, USH1F	AR	Compound Het	NM_033056.4:c.3668_3669delTT	**p.(Ile1223SerfsTer3)**	–							–		0/236	ND	ND	ND	Pathogenic
		***PCDH15***		AR	Compound Het	NM_033056.4:c.1737C>G	**p.(Tyr579Ter)**	–			DC	B			–	26	0/236	ND	0	0	Pathogenic
**29**	**II.3**	***USH2A***	USH2A	AR	Compound Het	NM_206933.3:c.13361T>A	**p.(Val4454Asp)**	–	D	PbD	DC	P	M	C	0.371	28.3		2/216	0.224	0.02	VUS
		***USH2A***		AR	Compound Het	NM_206933.3:c.5612G>A	**p.(Gly1871Asp)**	–	D	PbD	DC	P	M	C	0.663	25.2		0/216	0.371	0.105	VUS
*Variants found in genes associated with other syndromic forms of hearing loss*																					
**14**	**II.1**	***CHD7***	CHARGE syndrome, Hypogonadotropic hypogonadism 5 with or without anosmia	AD variable penetrance and expressivity	Het	NM_017780.4:c.8276A>G	**p.(Gln2759Arg)**	–	D	PsD	DC	P	M	C	0.236	26.9	0/236	ND	ND	ND	VUS
**16**	**II.1**	***CHD7***	CHARGE syndrome, Hypogonadotropic hypogonadism 5 with or without anosmia	AD variable penetrance and expressivity	Het	NM_017780.4:c.2613+5G>A		3.527			DC	Ph		C	–	14.35		0/216	0.027	0.007	VUS
**10**	**II.2, II.3**	***OPA1***	Optic atrophy plus syndrome	AD variable penetrance and expressivity	Het	NM_015560.2:c.2794C>T	**p.(Arg932Cys)**	–	D	PbD	DC	P	M		0.855	28.6	0/236	ND	0.008	0.002	VUS
**26**	**II.4**	***SPTLC1***	Neuropathy, hereditary sensory and autonomic type IA	AD variable penetrance	Het	NM_001368273.1:c.881C>T	**p.(Thr294Met)**	–	D	PbD	DC	P	L	C	0.775	33	0/236	ND	0	0	VUS

* Variants were classified according to the ACMG/AMP Guidelines for interpretation of sequence variants in HL genes [22] with two modifications. The genetic causes of HL have not yet been well characterized in the Yoruba (YRI) population, and the information regarding variant MAF in this population is still limited, so we did not exclude any variant based on their “high” MAF. PP3 criterion was applied even if the REVEL score was below 0.7, if at least two of the algorithms used predicted that the variant was damaging or likely damaging (Fig. 1). Further information regarding these variants is presented in Supplementary Table 4.

*ND* no record in the database, *AD* autosomal dominant, *AR* autosomal recessive, *Het* heterozygous, *Hom* homozygous, *B* benign, *C* conserved, *D* damaging, *DC* disease causing, *H* high, *L* low, *M* medium, *N* neutral, *P* pathogenic, *PbD* probably damaging, *Ph* pathogenic (high confidence), *PsD* possibly damaging, *Po* polymorphism, *T* tolerated.

CNVs were annotated using ANNOVAR, BioMart database, and bedtools to obtain genes, functions, and known disease associations [21]. Rare variants (MAF < 0.5%) were selected based on variant frequency data from the Database of Genomic Variants and gnomAD. Candidate CNVs were visually inspected with the CoNIFER plotting tool and were subsequently validated using Digital Droplet PCR (ddPCR, BioRad Laboratories, Hercules, CA, USA) [24] if deemed of interest. ddPCR was also used to confirm the absence of a deletion in *trans* when a damaging variant appeared to be homozygous and gDNA of one parent was not available.

## Results

### Study subjects and families

In the 56 families studied from Ibadan, Nigeria, a total of 60 (*N*) independent cases of HL were studied (Supplementary Table 1). All individuals with HL had bilateral congenital or prelingual-onset severe-to-profound HL and used sign language. The presence of pathogenic, likely pathogenic and VUS with predicted deleterious effects in genes reported to be associated with HL was investigated (Fig. 1).

### *GJB2* pathogenic variants are not prevalent in the Yoruba population of Nigeria

Sanger sequence analyses of *GJB2* in 60 independent cases with HL revealed in one subject a heterozygous pathogenic recessive variant that was previously reported (NM_004004.6:c.405delC, p.(Tyr136fsTer32)). One heterozygous previously reported VUS was also identified in the 5’ UTR of *GJB2* (c.−6T>A) in three individuals from three different families. We excluded by PCR testing the possibility that a second allele involving deletions at the *DFNB1A/B* locus [del(*GJB6*-D13S1830), del(*GJB6*-D13S1854), and del(chr13:19,837,344-19,968,698)] was in *trans* in any of these four subjects who carry heterozygous *GJB2* variants.

### Exome sequencing reveals high heterogeneity in potential causal variants in genes associated with hearing loss

ES was performed using gDNA samples from 67 individuals with HL and a subset of family members without HL (48). The region encoding microRNA *MIR96* was Sanger-sequenced, but no variants were identified. The small size of the families and the absence of reported consanguinity precluded linkage or homozygosity mapping studies. Lack of access to one or both parents in several families limited our ability to confirm that identified heterozygous variants were compound heterozygous.

**Fig. 2:**
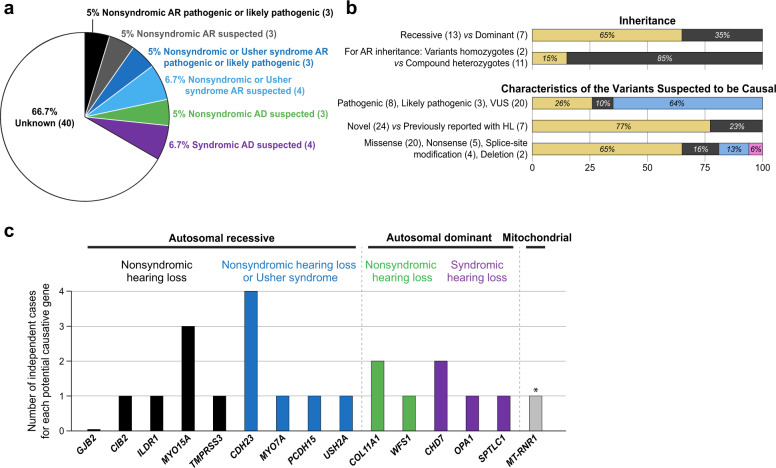
Etiology of hearing loss in a cohort of 56 Nigerian Yoruba families with 60 independent cases of hearing loss. **a** Relative distribution of the likely genetic causes of HL identified in Yoruba individuals according to ACMG/AMP criteria for interpretation of sequence variants in HL genes, mode of inheritance, and gene classification. AR autosomal recessive, AD autosomal dominant. The number of cases per category is indicated in parentheses. **b** Characteristics of the cases and variants identified. **c** Distribution of the cases as a function of the genes in which potential causative variants were identified. The associated modes of inheritance are indicated. *MT-RNR1 variant m.1555A>G was found in an individual with HL also carrying potential causative VUS in *CDH23* and *OTOF* (the latter were not indicated here but are available in Table 1).

Potential causative variants were found in 20 of 60 independent cases of HL of our cohort (Table 1 and Supplementary Table 4). These variants were identified in genes associated with nonsyndromic forms of HL in nine cases, in genes associated with nonsyndromic forms of HL or Usher syndrome in seven cases, and in genes associated with other forms of syndromic HL in four cases (Fig. 2a, c). Pathogenic or likely pathogenic variants were only identified in 6 of 20 independent cases of HL in our cohort (Table 1, Supplementary Table 4, and Fig. 2a). Sixteen percent of these variants were nonsense variants, 6% were small deletions, 13% were predicted to affect splicing, and 65% were missense variants (Fig. 2b). Seventy-seven percent of these variants had not been previously associated with HL. We also identified several potential CNVs. However, subsequent ddPCR experiments either did not confirm the presence of these CNVs or showed that they did not cosegregate with the HL.

### Variants identified in genes associated with nonsyndromic forms of hearing loss

**Fig. 3:**
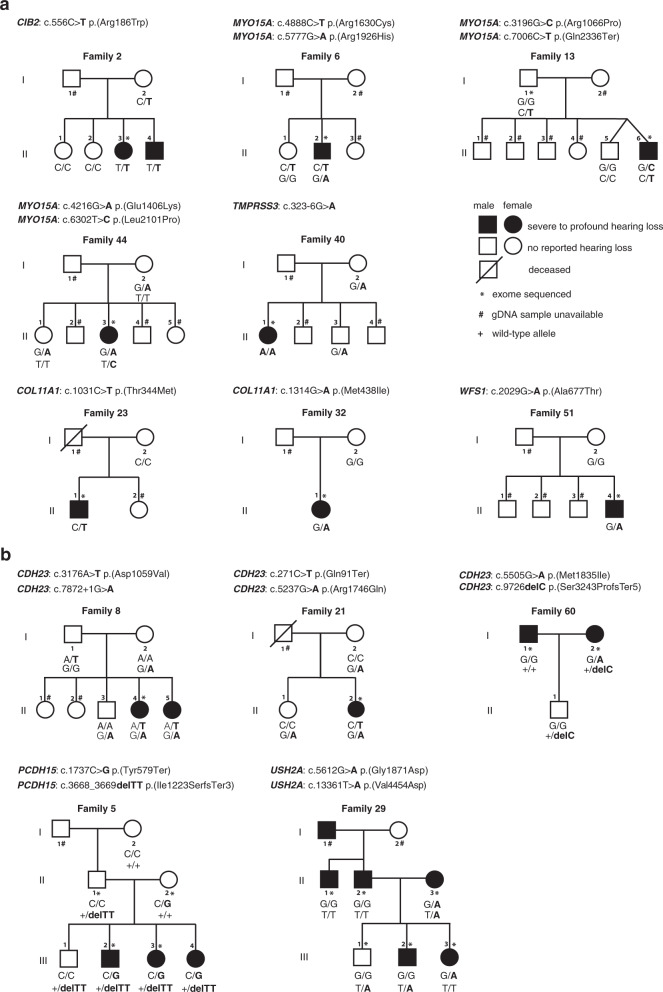
Pedigrees of Nigerian Yoruba families segregating likely causative variants in genes associated with nonsyndromic hearing loss and Usher syndrome. **a** Families with variants in genes associated with nonsyndromic hearing loss. **b** Families with variants in genes associated with nonsyndromic hearing loss or Usher syndrome. Circles represent females and squares indicate males. Solid symbols represent individuals with bilateral severe-to-profound HL; non-shaded symbols represent unaffected individuals; crossed symbols represent deceased individuals. Individuals studied by exome sequencing are indicated by an asterisk, # indicates individuals whose gDNA was not available. The genotype for the candidate variant(s) are shown above each pedigree. + represents the reference allele; /, variants in *trans*. Only pedigrees with segregation information are presented.

Variants predicted by bioinformatic analyses to be damaging and segregating with HL were identified in four genes known to be associated with recessive forms of nonsyndromic HL (Table 1, Supplementary Table 4, and Figs. 2 and 3a). A homozygous variant (confirmed by ddPCR) that is likely pathogenic was identified in *CIB2* [MIM: 605564, *DFNB48*] in Family 2. Potential compound heterozygous variants that are pathogenic or predicted to be damaging were detected in *ILDR1* [MIM: 609739, *DFNB42*] in Family 57 and in *MYO15A* [MIM: 602666, *DFNB3*] in Families 6, 13, and 44 (Table 1 and Fig. 3a). In addition, two damaging homozygous variants were identified in *TMPRSS3* [MIM: 605511, *DFNB8/B10*] (NM_024022.3:c.323-6G>A, a previously reported variant, and c.1363T>C, p.(Ter455ArgextTer9)) in one subject with HL in Family 40. Using ddPCR, we confirmed that this subject does not carry a deletion in this region, and further showed that these variants are present within a stretch of homozygosity at the telomeric ~5.3-Mb region of chromosome 21q. It may be a consequence of maternally inherited partial uniparental isodisomy or this individual with HL could have received the same region of chromosome 21 from her father, which we could not examine (gDNA not available). Analysis of the exome of this individual with HL did not reveal other regions of homozygosity, suggesting that she is not the result of a consanguineous mating.

Potential causative variants were identified in two genes associated with dominant forms of HL: in *COL11A1* [MIM: 120280, *DFNA37*] NM_001854.4: c.1031C>T, p.(Thr344Met) in Family 23, and NM_001854.4:c.1314G>A, p.(Met438Ile) in Family 32, and in *WFS1* [MIM: 606201, *DFNA6/A14/A38*] NM_006005.3:c.2029G>A, p.(Ala677Thr) in Family 51 (Table 1, Supplementary Table 4, and Fig. 3a). These variants were not inherited maternally and paternal gDNA was not available in these three families to assess whether these variants arose *de novo*. Pathogenic variants in *COL11A1* have been associated with nonsyndromic forms of HL *DFNA37* [MIM: 618533] [25], and syndromic HL associated with Marshall and Stickler Syndromes [MIM: 154780 and 604841] [26]. In Family 32, a second variant predicted to be damaging, NM_001854.4: c.4049C>G, p.(Ser1350Cys), was identified in the individual with HL (Table 1 and Supplementary Table 4). This variant was inherited from the mother who did not have HL. Further evaluation of this individual with HL for potential signs of Stickler Syndrome was not possible. Variants of *WFS1* are also associated with AD Wolfram-like Syndrome and AR Wolfram Syndrome [MIM: 614296 and 222300].

In these nine families, among these rare variants predicted to be deleterious by bioinformatic approaches, only the homozygous *CIB2* variant, *TMPRSS3* splice variant, and potential compound heterozygous *ILDR1* variants were classified as pathogenic or likely pathogenic according to ACMG/AMP guidelines for the interpretation of sequence variants in HL genes [22]. In the other families, one or both of the identified variants were classified as VUS (Table 1).

### Variants identified in genes associated with nonsyndromic forms of hearing loss or Usher syndrome

Variants predicted to be damaging and known or suspected to be compound heterozygous were identified in *CDH23* [MIM: 605516] in Families 8, 21, 56 (father with HL), and 60 (mother with HL), *MYO7A* [MIM: 276903] in Family 56 (mother with HL), *PCDH15* [MIM: 605514] in Family 5, and *USH2A* [MIM: 608400] in Family 29 (mother with HL) (Table 1, Supplementary Table 4, and Fig. 3b). In Family 60 (mother with HL), two VUS were found in *OTOF*, in addition to the two VUS identified in *CDH23* (Table 1). Overall, in these seven families with potential causal variants in genes associated with nonsyndromic HL or Usher syndrome [27, 28], the variants met both ACMG/AMP criteria for the interpretation of sequence variants in HL genes for pathogenic or likely pathogenic classification in three families (Families 5, 8, and 21).

### Variants found in genes associated with other syndromic forms of hearing loss

**Fig. 4:**
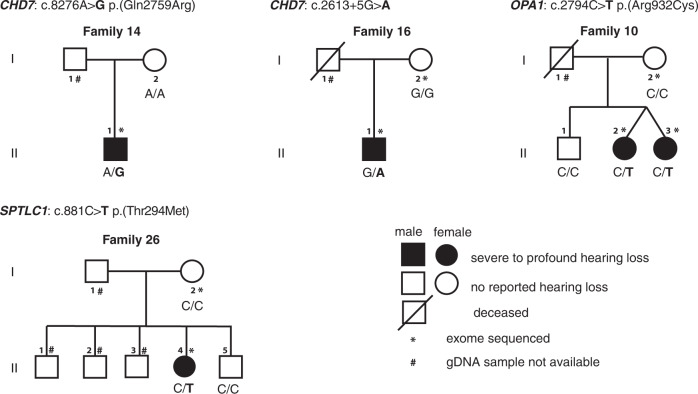
Pedigrees of Nigerian Yoruba families segregating likely causative variants in genes associated with syndromic forms of hearing loss. Features in addition to HL associated with pathogenic variants in these genes are known to have variable expressivity. Legend for pedigree drawings is similar to that of Fig. 3. The genotype for the candidate variant(s) are shown above each pedigree. + represents the reference allele; /, variants in *trans*.

We also analyzed our cohort for variants in 175 genes known to be involved in syndromic forms of HL (Supplementary Information 3), following the same strategy presented in Fig. 1. Variants predicted to be damaging were identified in *CHD7* [MIM: 608892] (Families 14 and 16), *OPA1* [MIM: 605290] (Family 10), and *SPTLC1* [MIM: 605712] (Family 26; Fig. 4). These variants, although classified as VUS, were all predicted to be damaging by multiple algorithms. The variants were absent in Yoruba controls and extremely rare in other populations (Table 1 and Supplementary Table 4). Pathogenic variants of *CHD7* are known to be associated with CHARGE syndrome [29] [MIM: 214800] and hypogonadotropic hypogonadism with or without anosmia [30] [MIM: 612370]. *OPA1* pathogenic variants have been reported in patients with AD optic atrophy 1 (ADOA, [MIM: 165500]), which can also be associated with delayed-onset sensorineural deafness (ADOAD) [MIM: 125250] [31, 32]. *SPTLC1* pathogenic variants have been reported in patients with hereditary sensory and autonomic neuropathy type IA [MIM: 162400] [33].

### A few rare mitochondrial variants were identified in *MT-RNR1*

We used Sanger sequencing to detect pathogenic variants in three mitochondrial genes reported to be associated with HL. We identified several variants in *MT-RNR1* [MIM: 561000] encoding the 12S rRNA (Supplementary Table 5). Eight of those variants were absent from Yoruba control samples and rare in other populations (Table 2). Although the pathogenicity of most of these variants is unknown, the variant m.1555A>G found in Family 60 (mother with HL) has previously been associated with aminoglycoside-induced HL as well as late-onset nonsyndromic HL not associated with aminoglycoside exposure [34–37]; however, other potential causes of HL have also been identified in the same individual (Table 1). We did not detect variants in *MT-TS1* [MIM: 590080]. One variant was found in *MT-TL1* [MIM: 590050]. It was predicted to be benign (m.3277G>A, c.48G>A) and was detected in the probands and their unaffected mothers in both Families 1 and 51. In Family 1, it was also detected in one unaffected sibling.

**Table 2:** Rare variants of *MT-RNR1* identified in individuals with hearing loss but absent from 118 YRI control mitochondrial genomes.

Variant position in mitochondria genome RefSeq NC_012920.1	dbSNP number	Conservation (45 species compared) from Mitomap	GenBank frequency in Mitomap based on 52,633 full length sequences (number of GenBank sequences in which it was found)	Family ID and carriers	Other likely cause of HL identified in this family	Previously reported
m.813A>G	rs878985110	22.22%	0.41% (216)	62 (I.2, II.1^b^)	No	Yes
m.955AC>A also known as m.956delC also known as m.960delC	rs111033185	N/A	0.10% (52)^a^	53 (I.2^b^, II.1)	No	Yes
m.955A>ACC also known as m.960C>CCC also known as m.956-960insCC	rs111033185	N/A	0.13% (66)^a^	45 (I.2^b^, II.1, II.2^b^)	No	Yes
m.959C>T	ND	11.11%	0.05% (24)	54 (I.2^b^, II.5, II.6)	No	Yes
m.961T>C, insCn	ND	N/A	ND	56 (I.2, II.1^b^)	Yes	Yes
m.1131C>T	ND	33.33%	ND	42 (I.2^b^, II.2)	No	No
m.1290C>T	rs1556422517	28.89%	0.05% (24)	26 (I.2^b^, II.4, II.5^b^)	Yes	Yes
m.1555A>G	rs267606617	86.67%	0.15% (78)	60 (I.2, II.1^b^)	Yes	Yes

Each variant was only detected in one family. Conservation values are based on the comparison of 45 species (Mitomap). Variant frequency values correspond to GenBank frequency in Mitomap based on 52,633 full length sequences.

The haplogroup lineage distribution of the Mitomap’s reference sequences is N: 67%, L: 12%, and M: 21%.

*ND* no record in the database, *HL* hearing loss, *ND* no record in the database, *N/A* not applicable.

^a^The frequency reported here corresponds to the sum of the frequencies reported for each way this variant was reported.

^b^Carrier without HL.

Overall, our molecular genetic analyses indicate a remarkably high level of heterogeneity associated with childhood HL in Yorubas from Ibadan, Nigeria, including the presence of the mitochondrial variant associated with aminoglycoside-induced HL m.1555A>G.

## Discussion

No prevalent pathogenic variants for HL were identified in this cohort of 56 small families of Yoruba ethno-lingual ancestry ascertained in or near Ibadan, Nigeria and segregating severe-to-profound bilateral sensorineural HL with congenital or early childhood onset. Only heterozygous variants of *GJB2* were identified, none of which were associated in *trans* with any of the three previously reported deletions in the genomic region of *GJB2-GJB6*. These results are consistent with those reported for 44 probands from Nigeria with nonsyndromic HL [15] and 90 families from Nigeria with nonsyndromic mild-to-profound prelingual HL [14]. In addition, none of the variants we identified were previously reported in those studies, supporting our conclusion of highly diverse genetic etiology of childhood HL in Nigeria. The heterogeneity of variants associated with HL may reflect the genomic heterogeneity of Sub-Saharan Africans and the fact that the Ibadan population has lived in a large city for many centuries with a constant flux of people. For AR forms of HL, homozygous variants were only found in a few cases, consistent with a lack of consanguineous matings in Yoruba population and the diverse array of variants identified at low frequency.

Different variants of a gene may cause either a syndromic form of HL such as Usher syndrome or nonsyndromic HL [7, 38]. Although our recruitment focused on probands with nonsyndromic HL, we identified several pathogenic, likely pathogenic and VUS with predicted deleterious effects in genes that can cause nonsyndromic HL or Usher syndrome (*CDH23*, *MYO7A*, *PCDH15*, and *USH2A*), CHARGE syndrome (*CHD7*), syndromic optic atrophy (*OPA1*), and a hereditary sensory and autonomic neuropathy (*SPTLC1*). The HL phenotype in these individuals may indeed be nonsyndromic, but a syndromic association could have been missed due to the young age of the patients at the time of examination, before the onset of signs or symptoms such as retinitis pigmentosa as the cause of vision loss in Usher syndrome. Furthermore, some of the signs and symptoms may have been present but either subclinical or may not have been detected at the time of examination. Another hypothesis is that there are modifiers present in African populations which are not present in the populations in which these syndromes have been phenotypically characterized. Variable expressivity of clinical presentations associated with *CHD7* and *OPA1* variants, both within and between families, has been previously documented [30, 39].

In a subset of families, including Family 60 that has a mother with HL, we identified several potential causes of HL. Lack of access to parental gDNA and limited information regarding the pathogenicity of the variants does not allow a definitive conclusion regarding the etiology of the HL in this family.

In 66.7% of the cases, the analyses presented here did not identify any potential causative variants in nonsyndromic and syndromic HL reported genes. This may be due to technical difficulties such as variants missed that are located in poorly sequenced GC rich coding exons, deletions of the scale of an exon and insertions that may not have been detected by our CNV analyses, failure to detect causal variants affecting splicing or in noncoding regions of the genome, unannotated exons for which there are no probes in the commercial ES reagents we used, novel HL genes or a nongenetic, multigenic, or multifactorial etiology of the HL in those families. We identified additional rare VUS with predicted deleterious effects in some of the unsolved families in this study in genes associated with syndromic forms of HL (Supplementary Table 3). Further clinical examinations of the individuals carrying such variants would be very informative, but are hampered by the difficulty of re-contacting and re-phenotyping the families. In numerous families, we identified rare heterozygous pathogenic or likely pathogenic variants in genes that are associated with recessive forms of HL. The presence of these variants could be coincidental but could also indicate that we failed to identify a second pathogenic variant responsible for the phenotype.

In conclusion, our study highlights the extreme heterogeneity of variants and genes associated with HL in the Yoruba population of Sub-Saharan Africa. This work underscores the need for comprehensive genomic sequencing approaches for molecular genetic diagnosis of HL in Sub-Saharan Africans.

## Supplementary information

Description of Supplementary Data, Supplementary Table 1, Supplementary Information 1–3

Supplementary Table 2

Supplementary Table 3

Supplementary Table 4

Supplementary Table 5

## Data Availability

All variants thought to cause HL have been submitted to the ClinVar public database under the reference SUB10099771 (https://www.ncbi.nlm.nih.gov/clinvar/).
