# Supplementary material for: Genomic analysis of childhood hearing loss in the Yoruba population of Nigeria
Source: Eur J Hum Genet. 2021 Nov 26;30(1):42–52. doi: 10.1038/s41431-021-00984-w (PMC8738750; doi:10.1038/s41431-021-00984-w)
Supplement: Supplementary file 1 — Description of Supplementary Data, Supplementary Table 1, Supplementary Information 1–3 [file 41431_2021_984_MOESM1_ESM.docx]

**Description of Supplemental Data**

Supplemental Data include 5 tables and 3 lists.

**Supplementary Table 1.** Reported composition and hearing status of the 56 families studied.

**Supplementary Table 2.** Primers used in this study are presented in multiple sheets.

**Supplementary Table 3.** List of variants identified in genes associated with nonsyndromic and syndromic forms of HL in the individuals with HL of our cohort*.*

**Supplementary Table 4.** Detailed information regarding the likely causative variants identified by ES in genes reported to be associated with HL presented in Table 1.

**Supplementary Table 5.** Summary of all the variants detected in *MT-RNR1* in the individuals with HL of our cohort.

**Supplementary Information 1.** List of URLs used in this study and corresponding references.

**Supplementary Information 2.** List of reported genes associated with nonsyndromic forms of HL considered in our analysis.

**Supplementary Information 3.** List of reported genes associated with syndromic forms of HL considered in our analysis.

**Supplementary Table 1.** Reported composition and hearing status of the 56 families studied.

Only children with available genomic DNA (gDNA) were included.

A total of 70 individuals with hearing loss were part of this cohort, 37 were females and 33 were males.

| **Group** | **Parent 1** | **Parent 2** | **Child** | **Sibling 1** | **Sibling 2** | **Sibling 3** | **Number of Families** |
| --- | --- | --- | --- | --- | --- | --- | --- |
| I.A | No HL | No HL | HL | - | - | - | 5 |
|  |  |  | HL | No HL | - | - | 8 |
|  |  |  | HL | HL | - | - | 1 |
|  |  |  | HL | HL | No HL | - | 2 |
|  |  |  | HL | HL | HL | No HL | 1 |
| I.B | No HL | No HL gDNA not available | HL | - | - | - | 12 |
|  |  |  | HL | No HL | - | - | 16 |
|  |  |  | HL | HL | No HL | - | 2 |
| I.C | No HL gDNA not available | No HL gDNA not available | HL | No HL | - | - | 1 |
| II.A | HL | HL | - | - | - | - | 1 |
|  |  |  | No HL | - | - | - | 2 |
|  |  |  | HL | HL | No HL | - | 1* |
| II.B | HL | HL  gDNA not available | - | - | - | - | 1 |
|  |  |  | No HL | - | - | - | 1 |
|  |  |  | No HL | No HL | - | - | 2 |

* The gDNA of a sibling with hearing loss of one of these parents with hearing loss was also available in this family.

HL, hearing loss; gDNA, genomic DNA.

In the 56 families studied from Ibadan, Nigeria, 48 families presented with unaffected parents: 42 presented with one affected child, five with two affected children, and one with three affected children. Both parents participated in 17 of these families, while only one parent participated in 30 families, and neither parent participated in one family. Both parents, unrelated to each other, had HL in eight families, but only twelve parents provided gDNA. One of these families had two children with HL and the other seven families did not have children with HL. Thus, a total of 60 (*N*) independent cases of HL was studied in our cohort.

**Supplementary Information 1.** List of URLs used in this study and corresponding references.

World Health Organization (WHO), <https://www.who.int/news-room/fact-sheets/detail/deafness-and-hearing-loss>

Hereditary Hearing Loss Homepage, <https://hereditaryhearingloss.org>

Online Mendelian Inheritance in Man (OMIM), <https://www.omim.org/>

*Annotation*

ANNOVAR (1), <http://annovar.openbioinformatics.org/>

Bcbio-nextgen germline variant calling pipeline, <https://bcbio-nextgen.readthedocs.io/en/latest/contents/pipelines.html#germline-variant-calling>

bedtools (2), https://bedtools.readthedocs.io/en/latest/

Biomart database (3), <http://www.biomart.org/>

Copy number inference from exome reads (CoNIFER) pipeline (4)

dbNSFP (5), <https://sites.google.com/site/jpopgen/dbNSF>

dbscSNV1.1 (6), <http://www.liulab.science/dbscsnv.html>

GATK (7), https://gatk.broadinstitute.org/hc/en-us

Ingenuity Variant Analysis, <https://digitalinsights.qiagen.com/products/ingenuity-variant-analysis/>

Kinship-based INference for Gwas (KING) algorithm (8)

plinkv1.9 (9), https://www.cog-genomics.org/plink/

*Genome browsers*

Ensembl, <https://www.ensembl.org/>

Human Genome Browser at UCSC, <https://genome.ucsc.edu/>

*Pathogenicity prediction*

Combined annotation dependent depletion (CADD) (10), <https://cadd.gs.washington.edu/>

FATHMM-MKL or XF (11, 12), <http://fathmm.biocompute.org.uk/>

Mutation Assessor (13), <http://mutationassessor.org/>

Mutation Taster (14), <http://www.mutationtaster.org/>

PolyPhen-2 (15), <http://genetics.bwh.harvard.edu/pph2/>

REVEL (16), https://sites.google.com/site/revelgenomics/

SIFT (17), <https://sift.bii.a-star.edu.sg/>

Varsome, <https://varsome.com/>

*Evolutionary conservation*

*PhyloP* (18)

Scores and interpretation also based on (19)

*Splicing*

BDGP Splice Site Prediction by Neural Network (20), <https://www.fruitfly.org/seq_tools/splice.html>

Human splice finder (21), <http://www.umd.be/HSF/>

MaxEntScan (22)

NetGene2 prediction server (23, 24), http://www.cbs.dtu.dk/dtucourse/cookbooks/nikob/exercises/gf2_netgene2.htm

*Mitochondria studies*

Mitomap, A Human Mitochondrial Genome Database. <https://www.mitomap.org/MITOMAP>

*Population frequency information*

1000 Genomes (25), <https://www.internationalgenome.org/>

ExAC (26), https://gnomad.broadinstitute.org/

Exome Variant Server, NHLBI GO Exome Sequencing Project (ESP), <http://evs.gs.washington.edu/EVS/>

genome aggregation database (gnomAD) (27), <https://gnomad.broadinstitute.org/>

*Variants databases*

*CHD7 variants,* [www.chd7.org](http://www.chd7.org)

ClinVar (28, 29), [https://www.ncbi.nlm.nih.gov/clinvar/](https://www.ncbi.nlm.nih.gov/clinvar/RCV000362197/)

Database of Genomic Variant, http://dgv.tcag.ca/dgv/app/

dbSNP (30), <https://www.ncbi.nlm.nih.gov/snp/>

Deafness Variation Database (DVD) (31), <http://deafnessvariationdatabase.org/>

The Human Gene Mutation Database (HGMD) (32), [http://www.hgmd.cf.ac.uk/](http://www.hgmd.cf.ac.uk/ac/index.php)

**Supplementary Information 2.** List of reported genes associated with nonsyndromic forms of hearing loss considered in our analysis, and organized depending on the reported mode of inheritance (see (33) – genes “refuted” (*GJB6, MYO1A (33), GJA1 (34)*) were not included, the genes classified as “definitive, strong, moderate and limited” evidence of association with hearing loss are underlined, “disputed” genes are indicated by an asterisk, additional genes reported on hereditaryhearingloss.org were also included in our analysis.) Note that some of these genes are known to be associated with both nonsyndromic and syndromic forms of hearing loss, or can underlie both dominant and recessive forms of hearing loss. Those were therefore included in several pipelines of analysis.

**Autosomal Dominant Inheritance (50 genes)**

*ACTG1*

*CCDC50*

*CD164*

*CEACAM16*

*COCH*

*COL11A1*

*COL11A2*

*CRYM*

*DIABLO*

*DIAPH1*

*DMXL2*

*ESPN*

*EYA4*

*GJB2*

*GJB3**

*GRHL2*

*GSDME*

*HOMER2*

*IFNLR1*

*KCNQ4*

*KITLG*

*LMX1A*

*MCM2*

*MIR96*

*MYH14*

*MYH9*

*MYO1C**

*MYO1F**

*MYO3A*

*MYO6*

*MYO7A*

*NLRP3*

*OSBPL2*

*P2RX2*

*PDE1C*

*PLS1*

*POU4F3*

*PTPRQ*

*REST*

*SIX1*

*SLC17A8*

*SLC44A4*

*TBC1D24*

*TECTA*

*TJP2*

*TMC1*

*TMTC2**

*TNC*

*TRRAP*

*WFS1*

**Autosomal Recessive Inheritance (77 genes)**

*ADCY1*

*BDP1*

*BSND*

*CABP2*

*CDC14A*

*CDH23*

*CEACAM16*

*CIB2*

*CLDN9*

*CLDN14*

*CLIC5*

*COL11A2*

*DCDC2*

*ELMOD3*

*EPS8*

*EPS8L2*

*ESPN*

*ESRP1*

*ESRRB*

*FAM65B*

*FOXI1**  Enlarged vestibular aqueduct

*GAB1*

*GIPC3* Progressive sensorineural hearing loss that can be associated with audiogenic seizures

*GJB2*

*GRAP*

*GRXCR1*

*GRXCR2*

*HGF*

*ILDR1*

*KARS*

*KCNJ10** Enlarged vestibular aqueduct

*KIAA1199**

*LHFPL5*

*LOXHD1*

*LRTOMT*

*MARVELD2*

*MET*

*MPZL2*

*MSRB3*

*MYO15A*

*MYO3A*

*MYO6*

*MYO7A*

*NARS2*

*OTOA*

*OTOF* Auditory synaptopathy

*OTOG*

*OTOGL*

*PCDH15*

*PDZD7*

*PJVK*

*PNPT1*

*PPIP5K2*

*PTPRQ*

*RDX*

*RIPOR2*

*ROR1* Hearing loss associated with common cavity inner ear malformations and auditory neuropathy

*S1PR2*

*SERPINB6*

*SLC22A4*

*SLC26A4* Enlarged vestibular aqueduct

*SLC26A5*

*SPNS2*

*STRC*

*SYNE4*

*TBC1D24*

*TECTA*

*TMC1*

*TMEM132E*

*TMIE*

*TMPRSS3*

*TPRN*

*TRIOBP*

*TSPEAR**

*USH1C*

*WBP2*

*WHRN*

**X-linked Inheritance (5 genes)**

*AIFM1*

*COL4A6*

*POU3F4*

*PRPS1*

*SMPX*

**Supplementary Information 3.** List of reported genes associated with syndromic forms of hearing loss considered in our analysis and organized depending on their reported mode of inheritance (see (33), additional genes reported on hereditaryhearingloss.org were also included in our analysis).

**Autosomal Dominant Inheritance (76 genes)**

*ACTB* Baraitser-Winter syndrome

*ACTG1* Baraitser-Winter syndrome

*AFF3* KINSSHIP syndrome (KIdney anomalies, Nievergelt/Savarirayan mesomelic dysplasia, Seizures, Hypertrichosis and Intellectual disability with Pulmonary involvement)

*AFF4* CHOPS syndrome

*ANKH* Craniometaphyseal dysplasia

*ARID1A* Coffin-Siris syndrome

*ARID1B* Coffin-Siris syndrome

*ARID2*  Coffin-Siris syndrome

*ATP1A2* Migraine, familial basilar and associated diseases

*ATP6V1B2* Deafness with onychondystrophy

*CDKN1C*  Beckwith-Wiedemann syndrome

*CHD7* CHARGE syndrome

*COL2A1* Stickler syndrome

*COL4A3* Alport syndrome

*COL4A4* Alport syndrome

*COL5A1* Ehlers-Danlos syndrome, classic type, 1

*COL11A1* Marshall and Stickler syndrome

*COL11A2* Otospondylomegaepiphyseal dysplasia A

*CREBBP* Rubinstein-Taybi syndrome 1 and other syndromes

*DIAPH1* Hearing loss and macrothrombocytopenia

*DIAPH3* Auditory neuropathy

*DLX5* Split hand/foot malformation with or without hearing loss

*DNMT1* DNMT1 methylopathy

*DPF2* Coffin-Siris syndrome 7

*DSPP* Dentinogenesis imperfecta

*DVL1* Robinow syndrome, autosomal dominant 2

*EDN3* Waardenburg syndrome type IV

*EDNRB* Waardenburg syndrome type IV

*EYA1* Branchio-oto-renal syndrome

*FGFR2* Apert syndrome, Crouzon syndrome and other syndromes

*FGFR3* Camptodactyly, Tall Stature, Scoliosis, and Hearing Loss Syndrome

LADD syndrome

Meunke syndrome

*FOXC1* Axenfled-Rieger syndrome

*GATA2*  Emberger syndrome

*GATA3*  Hypoparathyroidism, Sensorineural Hearing Loss, and renal dysplasia

*GJB2* Syndromic hearing loss

*GJB3*  Erythrokeratodermia variabilis

*GJB6* Hidrotic ectodermal dysplasia (Clouston syndrome)

*JAG1* Alagile syndrome; Deafness, congenital heart defects, and posterior embryotoxon

*KAT6B* Genitopatellar syndrome and Say-Barber-Biesecker variant of Ohdo syndrome

*MED13*

*MITF* Waardenburg syndrome type II

*MYH9* MYH9 related diseases

*NF2* Neurofibromatosis Type II (cancer predisposition syndrome – often associated with progressive HL)

*NIPBL*  Cornelia de Lange syndrome 1

*NLRP3*  Muckle-Wells syndrome

*OPA1* Optic atrophy I, Optic atrophy plus syndrome

*PAX3*  Waardenburg syndrome type I, type III

*PITX2* Axenfeld-Rieger Syndrome

*PMP22* Charcot-Marie-Tooth

*POLD1* Mandibular hypoplasia, deafness, progeroid features, and lipodystrophy syndrome

*POLR1D* Treacher Collins Syndrome

*PTPN11* LEOPARD syndrome 1

Noonan syndrome 1

*RAD21* Cornelia de Lange syndrome 4

*RPS26*  Diamond-Blackfan anemia 10

*RERE* Neurodevelopmental disorder with or without anomalies of the brain, eye, or heart

*SALL1* Townes-Brocks syndrome

*SALL4* SALL4-related disorders

*SEMA3E* CHARGE syndrome

*SIX1*  Branchiootorenal syndrome

*SIX5* Branchiootorenal syndrome

*SMC3* Cornelia de Lange syndrome 3

*SMARCA4* Coffin-Siris syndrome

*SMARCB1* Coffin-Siris syndrome

*SMARCE1* Coffin-Siris syndrome

*SOX9* Campomelic dysplasia with or without autosomal sex reversal, Acampomelic campomelic dysplasia

*SOX10*  Waardenburg syndrome type II, type IV

*SOX11*  Coffin-Siris syndrome

*SPTLC1* Neuropathy, hereditary sensory and autonomic, type IA

*TCOF1* Treacher Collins Syndrome

*TFAP2A* Branchiooculofacial syndrome

*THRB* Thyroid hormone resistance

*TP63* various disorders associated with high percentage of conductive forms of hearing loss

*TUBB4B* Leber congenital amaurosis

*WFS1*  Wolfram-like syndrome

Neonatal-onset diabetes, congenital sensorineural deafness, and congenital cataracts

*WS2B* Waardenburg syndrome, type 2B

*WS2C* Waardenburg syndrome, type 2C

**Autosomal Recessive Inheritance (100 genes)**

*ABHD5* Chanarin-Dorfman syndrome

*ABHD12* PHARC syndrome

*ADGRV1/VLGR1/GPR98* Usher syndrome type 2C

*ALMS1* Alstrom Syndrome

*ARSB* Mucopolysaccharidosis

*ATP6V1B1* Renal tubular acidosis with deafness

*BAPX1* Spondylo-megaepiphyseal-metaphyseal dysplasia

*BCS1L* Bjornstad Syndrome

*BSND* Bartter Syndrome

*BTD* Biotinidase deficiency

*CACNA1D* SA node dysfunction and deafness

*CDC14A*  Hearing impairment and infertile male syndrome

*CD151*  Nephropathy with pretibial epidermolysis bullosa and deafness

*CDH23* Usher syndrome type 1D

*CDKRAP2* Microcephaly 3

*CEP78* Cone-rod dystrophy and hearing loss

*CEP250* Cone-rod dystrophy and hearing loss 2

*CHSY1* Temtamy preaxial brachydactyly syndrome

*CIB2* Usher syndrome type 1

*CISD2* Wolfram syndrome

*CLPP* Perrault syndrome

*CLRN1* Usher syndrome type 3A

*COL4A3* Alport syndrome

*COL4A4* Alport syndrome

*COL9A1* Stickler syndrome

*COL9A2* Stickler syndrome

*COL9A3* Epiphyseal dysplasia, multiple, 3, with or without myopathy

*COL11A2* Otospondylomegaepiphyseal dysplasia B

*CYP27A1* Cerebrotendinous xanthomatosis

*DLX5* Split hand/foot malformation with or without hearing loss

*DUOX2* Thyroid dyshormonogenesis 6

*EDN3* Waardenburg syndrome type IV

*EDNRB* Waardenburg syndrome type IV

*ERAL1* Perrault syndrome

*ERCC2* Xeroderma pigmentosum

*ERCC3* Xeroderma pigmentosum

*FDXR*  Auditory neuropathy and optic atrophy

*FGF3*  LAMM syndrome

*FGFR3* Camptodactyly, Tall Stature, Scoliosis, and Hearing Loss Syndrome

*FOXI1* Syndromic hearing loss

*GPSM2* Chudley-McCullough syndrome

*HARS2* Perrault syndrome

*HOXA1* [Duane syndrome](http://www.childrenshospital.org/research/labs/engle-laboratory/neurogenetics-research/duane-syndrome)Type III

*HOXB1* Facial paresis

*HSD17B4* Perrault syndrome

*KCNE1* Jervell and Lange-Nielsen syndrome

*KCNJ10* SeSAME syndrome

*KCNQ1* Jervell and Lange-Nielsen syndrome

*LARS2*  Perrault syndrome

*LHX3*  Hypopituitarism with or without SNHL

*LRP2* Donnai-Barrow syndrome

*MAN1B1* Mental retardation, autosomal recessive 15

*MANBA* Beta-mannosidosis

*MASP1* 3MC syndrome

*MYO7A* Usher syndrome type 1B

*NPHP4* Senior-Loken syndrome 4, Nephronophthisis 4

*OPA1*  Behr syndrome

*PEX1,2,3,6,7,10,12,11B,13,14,16,19,26* Zellweger Spectrum disorder/Refsum

*PCDH15* Usher syndrome type 1

*PAX3*  Waardenburg syndrome type III

*PCDH15* Usher syndrome type 1F

*POLR1C* Treacher Collins Syndrome

*POLR1D* Treacher Collins Syndrome

*RAB23* Carpenter syndrome (conductive/sensorineural hearing loss)

*RNASET2* Leukoencephalopathy, cystic, without megalencephaly

*SANS* Usher syndrome type 1G

*SBF1*  Charcot-Marie-Tooth disease, type 4B3

*SLC12A1* Bartter Syndrome

*SLC19A2* Thiamine-responsive megaloblastic anemia syndrome

*SLC26A4* Pendred syndrome

*SLC29A3* Histiocytosis-lymphadenopathy plus syndrome

*SLC4A11* Corneal endothelial dystrophy and deafness (Harboyan syndrome)

*SLC52A2* Brown-Vialetto-Van Laere syndrome

*SLC52A3* Brown-Vialetto-Van Laere syndrome

*SLITRK6* Deafness and myopia

*SNAI2* Waardenburg syndrome type II

*SPATA5* Epilepsy, hearing loss, and mental retardation syndrome

*SPINK5* Netherton syndrome

*SUCLA2* Mitochondrial DNA depletion syndrome

*TBC1D24* DOORS/EIEE/NSHL

*THRB* Thyroid hormone resistance

*TWNK*  Perrault syndrome

*USH1C* Usher syndrome type 1C

*USH1G* Usher syndrome type 1G

*USH2A* Usher syndrome type 2A

*WFS1*  Wolfram-like syndrome

*WHRN*  Usher syndrome type 2D

*XPC* Xeroderma pigmentosum, group C (progressive HL)

**X-linked Inheritance (12 genes)**

*AIFM1*  Auditory neuropathy spectrum

*BCAP31*  Deafness, dystonia, and cerebral hypomyelination

*COL4A5*  Alport syndrome Dominant penetrance High in males, Low to Moderate in females

*GLA* Fabry Disease

*HDAC8* Cornelia de Lange syndrome 5

*NDP* Norrie disease

*PRPS1*  PRPS1 deficiency disorder, Arts syndrome, Charcot Marie Tooth

Phosphoribosylpyrophosphate synthetase superactivity

*RPS6KA3* Coffin-Lowry syndrome

*SMC1A* Cornelia de Lange syndrome 2

*TBL1X* Central hypothyroidism and hearing loss, Ocular albinism with late-onset sensorineural deafness

*TIMM8A* Mohr-Tranebjaerg syndrome

*WDR45* Neurodegeneration with brain iron accumulation 5

**References**

1. Wang K, Li M, Hakonarson H. ANNOVAR: functional annotation of genetic variants from high-throughput sequencing data. Nucleic Acids Research. 2010;38(16):e164-e.

2. Quinlan AR, Hall IM. BEDTools: a flexible suite of utilities for comparing genomic features. Bioinformatics. 2010;26(6):841-2.

3. Zhang J, Haider S, Baran J, Cros A, Guberman JM, Hsu J, et al. BioMart: a data federation framework for large collaborative projects. Database (Oxford). 2011;2011:bar038.

4. Krumm N, Sudmant PH, Ko A, O'Roak BJ, Malig M, Coe BP, et al. Copy number variation detection and genotyping from exome sequence data. Genome Res. 2012;22(8):1525-32.

5. Liu X, Wu C, Li C, Boerwinkle E. dbNSFP v3.0: A One-Stop Database of Functional Predictions and Annotations for Human Nonsynonymous and Splice-Site SNVs. Hum Mutat. 2016;37(3):235-41.

6. Jian X, Boerwinkle E, Liu X. In silico prediction of splice-altering single nucleotide variants in the human genome. Nucleic Acids Res. 2014;42(22):13534-44.

7. McKenna A, Hanna M, Banks E, Sivachenko A, Cibulskis K, Kernytsky A, et al. The Genome Analysis Toolkit: a MapReduce framework for analyzing next-generation DNA sequencing data. Genome Res. 2010;20(9):1297-303.

8. Manichaikul A, Mychaleckyj JC, Rich SS, Daly K, Sale M, Chen W-M. Robust relationship inference in genome-wide association studies. Bioinformatics. 2010;26(22):2867-73.

9. Chang CC, Chow CC, Tellier LC, Vattikuti S, Purcell SM, Lee JJ. Second-generation PLINK: rising to the challenge of larger and richer datasets. Gigascience. 2015;4:7.

10. Rentzsch P, Witten D, Cooper GM, Shendure J, Kircher M. CADD: predicting the deleteriousness of variants throughout the human genome. Nucleic Acids Res. 2019;47(D1):D886-D94.

11. Rogers MF, Shihab HA, Mort M, Cooper DN, Gaunt TR, Campbell C. FATHMM-XF: accurate prediction of pathogenic point mutations via extended features. Bioinformatics. 2018;34(3):511-3.

12. Shihab HA, Rogers MF, Gough J, Mort M, Cooper DN, Day IN, et al. An integrative approach to predicting the functional effects of non-coding and coding sequence variation. Bioinformatics. 2015;31(10):1536-43.

13. Reva B, Antipin Y, Sander C. Predicting the functional impact of protein mutations: application to cancer genomics. Nucleic Acids Res. 2011;39(17):e118.

14. Schwarz JM, Rodelsperger C, Schuelke M, Seelow D. MutationTaster evaluates disease-causing potential of sequence alterations. Nat Methods. 2010;7(8):575-6.

15. Adzhubei IA, Schmidt S, Peshkin L, Ramensky VE, Gerasimova A, Bork P, et al. A method and server for predicting damaging missense mutations. Nat Methods. 2010;7(4):248-9.

16. Ioannidis NM, Rothstein JH, Pejaver V, Middha S, McDonnell SK, Baheti S, et al. REVEL: An Ensemble Method for Predicting the Pathogenicity of Rare Missense Variants. Am J Hum Genet. 2016;99(4):877-85.

17. Kumar P, Henikoff S, Ng PC. Predicting the effects of coding non-synonymous variants on protein function using the SIFT algorithm. Nat Protoc. 2009;4(7):1073-81.

18. Pollard KS, Hubisz MJ, Rosenbloom KR, Siepel A. Detection of nonneutral substitution rates on mammalian phylogenies. Genome Res. 2010;20(1):110-21.

19. Sun H, Yu G. New insights into the pathogenicity of non-synonymous variants through multi-level analysis. Sci Rep. 2019;9(1):1667.

20. Reese MG, Eeckman FH, Kulp D, Haussler D. Improved splice site detection in Genie. J Comput Biol. 1997;4(3):311-23.

21. Desmet FO, Hamroun D, Lalande M, Collod-Beroud G, Claustres M, Beroud C. Human Splicing Finder: an online bioinformatics tool to predict splicing signals. Nucleic Acids Res. 2009;37(9):e67.

22. Yeo G, Burge CB. Maximum entropy modeling of short sequence motifs with applications to RNA splicing signals. J Comput Biol. 2004;11(2-3):377-94.

23. Brunak S, Engelbrecht J, Knudsen S. Prediction of human mRNA donor and acceptor sites from the DNA sequence. J Mol Biol. 1991;220(1):49-65.

24. Hebsgaard SM, Korning PG, Tolstrup N, Engelbrecht J, Rouze P, Brunak S. Splice site prediction in Arabidopsis thaliana pre-mRNA by combining local and global sequence information. Nucleic Acids Res. 1996;24(17):3439-52.

25. Sudmant PH, Rausch T, Gardner EJ, Handsaker RE, Abyzov A, Huddleston J, et al. An integrated map of structural variation in 2,504 human genomes. Nature. 2015;526(7571):75-81.

26. Lek M, Karczewski KJ, Minikel EV, Samocha KE, Banks E, Fennell T, et al. Analysis of protein-coding genetic variation in 60,706 humans. Nature. 2016;536(7616):285-91.

27. Karczewski KJ, Francioli LC, Tiao G, Cummings BB, Alfoldi J, Wang Q, et al. The mutational constraint spectrum quantified from variation in 141,456 humans. Nature. 2020;581(7809):434-43.

28. Landrum MJ, Chitipiralla S, Brown GR, Chen C, Gu B, Hart J, et al. ClinVar: improvements to accessing data. Nucleic Acids Res. 2020;48(D1):D835-D44.

29. Landrum MJ, Lee JM, Benson M, Brown G, Chao C, Chitipiralla S, et al. ClinVar: public archive of interpretations of clinically relevant variants. Nucleic Acids Res. 2016;44(D1):D862-8.

30. Sherry ST, Ward MH, Kholodov M, Baker J, Phan L, Smigielski EM, et al. dbSNP: the NCBI database of genetic variation. Nucleic Acids Res. 2001;29(1):308-11.

31. Azaiez H, Booth KT, Ephraim SS, Crone B, Black-Ziegelbein EA, Marini RJ, et al. Genomic Landscape and Mutational Signatures of Deafness-Associated Genes. Am J Hum Genet. 2018;103(4):484-97.

32. Stenson PD, Mort M, Ball EV, Evans K, Hayden M, Heywood S, et al. The Human Gene Mutation Database: towards a comprehensive repository of inherited mutation data for medical research, genetic diagnosis and next-generation sequencing studies. Hum Genet. 2017;136(6):665-77.

33. DiStefano MT, Hemphill SE, Oza AM, Siegert RK, Grant AR, Hughes MY, et al. ClinGen expert clinical validity curation of 164 hearing loss gene-disease pairs. Genet Med. 2019;21(10):2239-47.

34. Paznekas WA, Boyadjiev SA, Shapiro RE, Daniels O, Wollnik B, Keegan CE, et al. Connexin 43 (GJA1) mutations cause the pleiotropic phenotype of oculodentodigital dysplasia. Am J Hum Genet. 2003;72(2):408-18.
